# Ultra-processed food consumption and indicators of obesity in the United Kingdom population (2008-2016)

**DOI:** 10.1371/journal.pone.0232676

**Published:** 2020-05-01

**Authors:** Fernanda Rauber, Eurídice Martínez Steele, Maria Laura da Costa Louzada, Christopher Millett, Carlos Augusto Monteiro, Renata Bertazzi Levy

**Affiliations:** 1 Núcleo de Pesquisas Epidemiológicas em Nutrição e Saúde, Universidade de São Paulo, São Paulo, Brazil; 2 Departamento de Nutrição, Faculdade de Saúde Pública, Universidade de São Paulo, São Paulo, Brazil; 3 Public Health Policy Evaluation Unit, School of Public Health, Imperial College London, London, England, United Kingdom; 4 Departamento de Medicina Preventiva, Faculdade de Medicina FMUSP, Universidade de São Paulo, São Paulo, Brazil; McMaster University, CANADA

## Abstract

We examined the association between the consumption of ultra-processed foods and adiposity in a nationally representative sample of the UK adult population. We studied 6,143 participants (19 to 96 years, 51.6% female) sampled by the UK National Diet and Nutrition Survey (2008–16). Food items reported in four-day food diary were classified according to the NOVA system. Multiple linear and logistic regressions were used to evaluate associations between the dietary contribution of ultra-processed foods (sex-specific quartile and continuous) and Body Mass Index (BMI), Waist Circumference (WC) and obesity (BMI>30kg/m^2^) and abdominal obesity (men: WC≥102cm, women: WC≥88cm) status. Models were adjusted for sociodemographic and lifestyle characteristics. In multivariable analyses, the highest consumption of ultra-processed food was associated with 1.66 kg/m^2^ higher BMI (95%CI 0.96–2.36), 3.56 cm greater WC (95%CI 1.79–5.33) and 90% higher odds for being obese (OR = 1.90, 95%CI 1.39–2.61), compared with the lowest consumption. A 10% increase in the consumption of ultra-processed foods was associated with an increase of 0.38 kg/m^2^ in BMI (95%CI 0.20–0.55), 0.87 cm in WC (95%CI 0.40–1.33) and 18% higher odds of being obese (OR = 1.18, 95%CI 1.08–1.28). The consumption of ultra-processed food was associated with an increase in BMI, WC and prevalence of obesity in both sexes. A dose response relationship was observed in both sexes, with a 10% increase in the consumption of ultra-processed foods being associated with a 18% increase in the prevalence of obesity in men and a 17% increase in women. Higher consumption of ultra-processed food is associated with greater adiposity in the UK adult population. Policy makers should consider actions that promote consumption of unprocessed or minimally processed foods and reduce consumption of ultra-processed foods.

## Introduction

Increasing prevalence of obesity is driving greater chronic disease and mortality burdens globally [1]. In the United Kingdom (UK), more than a quarter of adults are obese [2]. Concomitantly, ultra-processed food production and consumption are steadily increasing worldwide [3,4]. These products have led to displacement of dietary patterns based on minimally processed foods and freshly prepared meals [5,6]. In a recent analysis of the global trend of ultra-processed foods, the UK had the third highest volume sales of ultra-processed foods per capita (140.7 kg/capita/year) when compared to 80 high- and middle-income countries [7]. Analyses of 2008–2014 national dietary survey reveal that ultra-processed foods already make up more than half of the total dietary energy consumed in the UK [8].

Ultra-processed foods are industrial formulations of food-derived substances (e.g. oils, fats, sugars, starch, protein isolates) that contain little if any whole food and often include ingredients not used in culinary preparations, such as flavourings, colourings, emulsifiers and other cosmetic additives [9]. These formulations are hyper-palatable, attractive, convenient, sold in large packages, and highly marketed [6,10]. Analyses of nationally representative studies conducted in the UK [8], the United States [11], Canada [12] and Brazil [13] have shown strong associations between consumption of ultra-processed foods and dietary nutrient profiles that predict increased risk of obesity and several other diet-related chronic diseases. Moreover, associations between ultra-processed food intake and incidence of diet-related NCDs [14–16], including obesity [17], have been identified in several large prospective cohort studies. A cross-over, randomized controlled trial of healthy adults has shown that diets high in ultra-processed foods increased energy intake, body weight and fat gain, compared with a control diet matched in calories, sugar, fibre, and macronutrients [18].

Cross-sectional studies also have found association between ultra-processed food consumption and obesity. An ecological study across 19 European countries, including UK, found a positive association between national household availability of ultra-processed foods and national prevalence of obesity among adults [19]. Nationally-representative individual-based studies have found cross-sectional associations between the dietary share of ultra-processed foods and obesity among adults in Brazil [20], the United States [21], and Canada [22]. One study using data from the cycles 1–4 (2008–2012) of UK National Diet and Nutrition Survey (NDNS) found no association with body weight but this may reflect that relative energy intake from ultra-processed and processed foods was grouped together [23], as previous research carried out in Brazil found no association between increased consumption of processed foods and obesity [24].

Given the very high levels of ultra-processed food consumption and obesity in the UK, this study used more recent data from the UK NDNS (2008–2016) to evaluate the association between ultra-processed food consumption and a wider set of obesity indicators than previously studied—overall and abdominal obesity, greater body mass index and waist circumference.

## Methods

### Study design and population

The data source for this study were the National Diet and Nutrition Survey Rolling Programme (NDNS), which is a continuous cross-sectional survey of dietary habits and nutrient intakes of a representative sample of people living in private households in the UK. The current analysis used data collected between February 2008 and August 2016 (years 1–8 combined).

The sample was drawn from a selection of postcodes across the UK. Participants completed a consecutive four-day food diary and participated in an interview to collect background data that included data on demographics, socio-economic characteristics and health behaviours. Participants who completed the food diary on at least 3 days were subsequently visited by a nurse who conducted anthropometric measurements and collected a blood sample. The details of this survey have been previous described [25].

For this study, we included all participants aged 19 years or above (n = 6,155; age range 19 to 96 years). We excluded individuals who had all days of food diary classified as outliers (n = 10). We defined extreme total energy intake outliers as values above the 99th or below the 1st percentiles [26], for each food diary day and age group. No pregnant and breastfeeding women were included. In total, 6,143 participants were eligible for inclusion in the analyses and more than 96% completed the four food diary days.

Datafiles for the present study were obtained under license from the UK Data Archive (http://www.esds.ac.uk). All relevant research ethics and governance committees approved the survey.

### Dietary assessment

Participants were provided with written instructions and asked to record all foods and drinks consumed both at home and outside. Diary days were randomly selected to guarantee balanced representation of all days of the week. The diaries were coded using the in-house dietary assessment software, Diet In Nutrients Out (DINO), with nutrient values provided by the Department of Health’s Nutrient Databank, updated for each survey year [27,28].

We classified all recorded food items according to NOVA food classification system, which considers the physical, biological and chemical methods used during the food manufacturing process [9]. NOVA classifies foods into four groups: 1) unprocessed or minimally processed foods (e.g. fresh, dry or frozen fruits or vegetables; grains, flours and pasta; pasteurized or powder milk, plain yogurt, fresh or frozen meat); 2) processed culinary ingredients (e.g. table sugar, oils, butter, and salt); 3) processed foods (e.g. vegetables in brine, cheese, simple breads, fruits in syrup, canned fish); and 4) ultra-processed foods (e.g. soft drinks, sweet or savoury packaged snacks, confectionery; packaged breads and buns; reconstituted meat products and pre-prepared frozen or shelf-stable dishes)—the focus of this study. More information regarding the NOVA classification can be found elsewhere [9].

We classified foods by considering the NDNS variables ‘Food Number’ and ‘Subsidiary food groups’. When foods were judged to be homemade dishes, we applied the classification to the underlying ingredients in order to ensure more accurate classification. The NDNS database was provided with most food items systematically disaggregated into their individual components and the method adopted to disaggregate the food codes has been described in a previous paper [29]. Despite this, a few composite dishes were not disaggregated into constituent ingredients (less than 4%). In these cases, homemade dishes were categorised according to the main constituent ingredient. Details of how food item classification was undertaken are further explained in previously published papers [8,30].

We used the mean of all available days of food diary for each person to estimate the dietary contribution of ultra-processed foods (as a percentage of total energy intake).

### Outcomes assessment

Body Mass Index (BMI) and Waist Circumference (WC) were treated as continuous variables and used to generate measures of obesity and abdominal obesity. Height and weight were measured using a portable stadiometer and weighing scales by trained fieldworkers. WC was measured at the midpoint between the iliac crest and the lower rib to the nearest 0.1 centimetre. BMI was calculated by dividing weight by height squared (kg/m^2^) and values of ≥30 kg/m^2^ were classified as obesity according to World Health Organization criteria [31]. Abdominal obesity was defined as a WC ≥102 cm for men and ≥88 cm for women [32].

### Covariates

Covariates included were age in years, sex, ethnicity (white, non white), region (England North, England Central/Midlands, England South (including London), Scotland, Wales, and Northern Ireland), social class (routine & manual occupations, intermediate occupations, lower managerial & professional occupations, and higher managerial & professional occupations), survey year (years 1–8), physical activity (h/day of moderate or vigorous physical activity), smoking status (yes or no), sleep duration (h/day), and following a diet to lose weight (yes or no). Multiple imputation by chained equations was used to attribute numerical values to physical activity (15.1%), sleep duration (3.4%), social class occupation (3.1%), ethnicity (0.1%), and smoking (0.05%). As predictive variables, we considered sex, age, region, survey year, obesity status and ultra-processed food consumption. The data were imputed ten times, and the results exhibited satisfactory statistical reproducibility according to Monte Carlo error analysis [33].

### Statistical analysis

First, we estimated the distribution of total dietary energy intake according to NOVA food groups and subgroups. Then we examined how the mean energy share of ultra-processed foods and prevalence of obesity and high waist circumference (abdominal obesity) varied according to characteristics of the UK population. Differences in the exposure and outcomes by each individual characteristic were evaluated using bivariate linear regressions.

Multiple linear regression analysis was used to evaluate associations between the consumption of ultra-processed foods and BMI (kg/m^2^) and WC (cm), respectively. The normal probability plot of the residuals was constructed to verify the assumption of normality. Multiple logistic regression analysis was used to determine associations between the consumption of ultra-processed foods and the odds of obesity and abdominal obesity, respectively. We used the relative contribution of ultra-processed foods to total energy intake, both categorized into quartiles (using sex specific cut-offs) and continuous (10% increase in relative intake of ultra-processed foods), as the explanatory variables. For all outcomes, we first fitted a crude model and thereafter a multivariable model adjusted for 1) age, sex, ethnicity, region, survey year, and social class occupation, 2) also including physical activity, smoking, sleep duration and following a diet to lose weight. Test of linear trend was performed by treating quartile of ultra-processed food as an ordinal variable. Analyses were also performed in men and women separately, since previous studies had shown a potential difference between sexes in the association between ultra-processed food consumption and obesity [20,21].

Additional adjustment for total energy intake and consumption of fruits and vegetables were performed in all multivariable analyses.

NDNS study weights were used in all analyses to account for sampling and non-response error. All statistical analyses were carried out using Stata Statistical Software version 14. The p values reported were two-tailed, and a p value of <0.05 was considered statistically significant.

## Results

The distribution of total daily energy intake according to NOVA groups and subgroups is presented in Table 1. UK adults aged 19 years or over consumed on average 7,631kj/day (1,823 kcal SE 11.6), 54.3% of which came from ultra-processed foods, 30.7% from unprocessed or minimally processed foods, 10.1% from processed foods, and 4.9% from culinary ingredients.

**Table 1:** Distribution of total energy intake according to NOVA food groups. UK population aged 19 years or over (2008–16).

NOVA food groups	% of total energy intake *mean (SE)*
**Unprocessed or minimally processed foods**	**30.7 (0.3)**
Milk and plain yoghurt	4.6 (0.1)
Potatoes and other tubers and roots	3.6 (0.1)
Fruits	3.4 (0.1)
Red meat	3.4 (0.1)
Poultry	2.8 (0.1)
Cereals ^a^	2.6 (0.1)
Pasta	1.6 (0.1)
Eggs	1.6 (0.1)
Vegetables	1.7 (0.0)
Fresh fruit juice ^b^	1.0 (0.1)
Fish	1.4 (0.1)
Legumes	0.7 (0.0)
Other unprocessed or minimally processed foods ^c^	2.3 (0.1)
**Processed culinary ingredients**	**4.9 (0.1)**
Table sugar	1.9 (0.1)
Butter ^d^	1.7 (0.1)
Plant oil	0.8 (0.0)
Other processed culinary ingredients ^e^	0.4 (0.0)
**Processed foods**	**10.1 (0.2)**
Beer and wine	4.4 (0.2)
Cheese	3.0 (0.1)
Vegetables and other plant foods preserved in brine	1.0 (0.0)
Processed breads	0.8 (0.1)
Parma ham and other salted, smoked or canned meat or fish	0.6 (0.0)
Other processed foods ^f^	0.3 (0.0)
**Ultra-processed foods**	**54.3 (0.4)**
Ultra-processed breads	11 (0.2)
Packaged pre-prepared meals ^g^	7.8 (0.2)
Breakfast cereals	4.2 (0.1)
Sausage and other reconstituted meat products	3.6 (0.1)
Confectionary	3.2 (0.1)
Biscuits	3.2 (0.1)
Pastries, buns, and cakes	3.2 (0.1)
Industrial chips (French fries)	2.5 (0.1)
Soft drinks, fruit drinks and fruit juices	2.1 (0.1)
Milk-based drinks	2.0 (0.1)
Packaged salty snacks	1.6 (0.1)
Industrial pizza	1.5 (0.1)
Margarine and other spreads	2.1 (0.1)
Sauces, dressing and gravies	2.2 (0.1)
Industrial desserts	0.9 (0.1)
Other ultra-processed foods ^h^	3.1 (0.1)
**Total**	**100**

^a^ Including grains and flours.

^b^ Including UHT or pasteurised, and smoothies.

^c^ Including coffee, tea, sea foods, fungi, nuts, freshly prepared dishes based on one or more unprocessed or minimally processed food.

^d^ Including lard and suet shredded.

^e^ Including starches, coconut and milk cream, gelatin powder, vinegar.

^f^ Including salted, sweetened or oil roasted nuts or seeds, and condensed milk.

^g^ Including frozen and shelf-stable dishes and canned soups.

^h^ Including baked beans, meat alternatives, soy and others drinks as milk substitutes, and distilled alcoholic drink.

The consumption of ultra-processed foods (% of total energy) and the prevalence of obesity and abdominal obesity according to characteristics of the UK population are presented in Table 2. Ultra-processed food consumption was higher among men, those with white British ethnicity and smokers, but lower with increasing age and in higher social class groups. The prevalence of obesity was 26.4%, which increased with age and among those on a diet. Obesity prevalence was lower among higher social class groups and among those with higher physical activity and sleep duration levels. The prevalence of abdominal obesity was 40.7%, which was higher among woman, those with white British ethnicity and among those on a diet. Prevalence of abdominal obesity increased with age but was lower among higher social class groups and among those with higher physical activity and sleep duration levels.

**Table 2:** Consumption of ultra-processed foods (% of total energy) and prevalence of obesity and abdominal obesity according to characteristics of the UK population aged 19 years or over (NDNS, 2008–16).

	Distribution *(%)*	% of total energy intake from ultra-processed foods *mean (SE)*	Prevalence of, *% (SE)*	
			Obesity^a^	Abdominal obesity^b^
**Sex**				
Male	48.4	55.9 (0.6)	25.6 (1.5)	35.0 (1.6)
Female	51.6	52.8* (0.4)	27.1 (1.3)	46.1* (1.5)
**Age group**				
19 to 29 years	18.7	59.2 (1.3)	15.5 (2.4)	19.2 (2.5)
30 to 59 years	51.0	54 (0.4)	27.5 (1.4)	40.1 (1.5)
≥60 years	30.3	51.8^α^ (0.5)	31.5^α^ (1.8)	55.3^α^ (1.8)
**Ethnicity**				
White	89.5	55.4 (0.4)	26.7 (1.0)	40.7 (1.2)
Non-white	10.5	45.4* (1.2)	23.4 (3.5)	30.5* (3.7)
**Region**				
England North	23.2	56.1 (0.7)	29.4 (2.3)	43.9 (2.3)
England Central/Midlands	16.6	56.6 (1.0)	31.6 (2.6)	43.2 (2.9)
England South (including London)	43.9	51.7* (0.6)	21.9* (1.9)	36.1* (1.6)
Scotland	8.6	56.5 (1.1)	27.9 (2.1)	44.3 (3.9)
Wales	4.9	55.0 (1.0)	29.8 (2.4)	46.8 (3.0)
Northern Ireland	2.8	58.7* (0.8)	29.1 (2.4)	50.6 (3.2)
**Social class occupation**				
Routine & manual occupations	36.3	57.3 (0.7)	30.3 (1.8)	44.5 (2.0)
Intermediate occupations	19.1	53.4 (0.8)	27.9 (2.3)	39.5 (2.5)
Lower managerial & professional occupations	26.0	53.8 (0.7)	24.1 (1.9)	36.0 (2.1)
Higher managerial & professional occupations	18.7	50.3^α^ (0.7)	20.2^α^ (2.1)	36.6^α^ (2.6)
**Physical activity** ^c^				
First quartile	22.1	55.5 (0.7)	32.2 (2.3)	52.2 (2.5)
Second quartile	27.8	53.3 (0.8)	26.7 (2.0)	38.9 (2.1)
Third quartile	25.9	53.5 (0.7)	21.7 (1.8)	34.8 (2.2)
Fourth quartile	24.2	55.4 (0.7)	25.7^α^ (2.1)	34.4^α^ (2.2)
**Smoking status**				
Non-smoker	78.9	53.3 (0.4)	27.0 (1.1)	40.6 (1.2)
Smoker	21.1	58.3* (0.9)	23.9 (2.4)	36.1 (2.6)
**Sleep duration**				
<6 h/day	33.1	54.7 (0.6)	32.2 (1.8)	45.6 (2.0)
7–8 h/day	35.7	53.9 (0.5)	23.0 (1.6)	35.0 (1.8)
>8 h/day	31.2	54.4 (0.8)	24.0* (1.8)	38.8* (2.1)
**Following a special diet**				
No	87.9	54.5	22.7 (1.0)	37.8 (1.1)
Yes	12.1	52.9	53.0 (3.2)*	62.3 (3.2)*
**Total**	**100**	**54.3 (0.4)**	**26.4 (1.0)**	**40.7 (1.1)**

^a^ Defined as Body Mass Index ≥30 kg/m^2^ (World Health Organization, 2003).

^b^ Defined as waist circumference ≥102/88 cm for men and women, respectively (World Health Organization, 2008).

^c^ Time spent at moderate or vigorous physical activity (h/d).

* p<0.05 (first category as reference)

^α^ P<0.05 for linear trend across categories

Crude and adjusted analyses of the association between the dietary contribution of ultra-processed foods and adiposity are shown in Table 3. The consumption of ultra-processed foods ranged from ~35% (1st quartile) to ~74% (4th quartile) of total energy intake. The crude prevalence of obesity increased from 22.8% in the first to 31.6% in the fourth quartile of ultra-processed food consumption; while the crude prevalence of abdominal obesity was similar across quartiles (from 41.2% to 41.7%, respectively). In multivariable analyses, the highest consumption of ultra-processed food was associated with 1.66 kg/m^2^ higher BMI (95%CI 0.96–2.36), 3.56 cm greater WC (95%CI 1.79–5.33) and 90% higher odds for being obese (OR = 1.90, 95%CI 1.39–2.61), compared with the lowest consumption. A significant linear trend was observed for the association between quartile of ultra-processed food consumption and the three outcomes (P<0.001). In the full adjusted model, a 10% increase in the consumption of ultra-processed foods was associated with an increase of 0.38 kg/m^2^ in BMI (95%CI 0.20–0.55), 0.87 cm in WC (95%CI 0.40–1.33) and 18% in the odds of obesity (continuous OR = 1.18, 95%CI 1.08–1.28). No association was observed for odds of abdominal obesity.

**Table 3:** Crude and adjusted analyses of the association between the dietary contribution of ultra-processed food and indicators of obesity among the UK population aged 19 years or over (NDNS, 2008─16).

	Consumption of ultra-processed foods (% of total energy)					
	Sex-specific quartile ^a^				p for trend ^α^	Continuous (10% increase in the consumption)
	1	2	3	4		
**BMI (kg/m**^**2**^**)**						
Mean (SE)	26.9 (0.2)	27.1 (0.2)	27.2 (0.3)	28.0 (0.3)		
Crude, *coef (95% CI)*	0	0.27 (-0.33; 0.87)	0.33 (-0.38; 1.04)	1.15 (0.37; 1.94)	0.006	0.29 (0.10; 0.48)
Model 1^b^, *coef (95% CI)*	0	0.25 (-0.33; 0.83)	0.33 (-0.33; 0.99)	1.47 (0.73; 2.21)	<0.001	0.35 (0.16; 0.53)
Model 2^c^, *coef (95% CI)*	0	0.39 (-0.15; 0.94)	0.35 (-0.27; 0.97)	1.66 (0.96; 2.36)	<0.001	0.38 (0.20; 0.55)
**Waist circumference (cm)**						
Mean (SE)	91.6 (0.6)	92.6 (0.6)	92.5 (0.8)	94.0 (0.9)		
Crude, *coef (95% CI)*	0	1.01 (-0.68; 2.70)	0.89 (-0.99; 2.77)	2.43 (0.33; 4.53)	0.034	0.79 (0.26; 1.31)
Model 1^b^, *coef (95% CI)*	0	0.26 (-1.22; 1.74)	0.72 (-0.93; 2.37)	3.52 (1.66; 5.39)	0.001	0.80 (0.31; 1.28)
Model 2^c^, *coef (95% CI)*	0	0.55 (-0.87; 1.98)	0.42 (-1.16; 2.01)	3.56 (1.79; 5.33)	<0.001	0.87 (0.40; 1.33)
**Obesity**^d^						
Prevalence (SE)	22.8 (1.8)	24.7 (1.9)	27.3 (2.1)	31.6 (2.4)		
Crude, *OR (95% CI)*	1	1.11 (0.83; 1.48)	1.27 (0.95; 1.70)	1.56 (1.16; 2.11)	0.002	1.13 (1.05; 1.21)
Model 1^b^, *OR (95% CI)*	1	1.11 (0.83; 1.50)	1.26 (0.94; 1.71)	1.71 (1.25; 2.33)	0.001	1.15 (1.06; 1.25)
Model 2^c^, *OR (95% CI)*	1	1.19 (0.88; 1.61)	1.31 (0.97; 1.78)	1.90 (1.39; 2.61)	<0.001	1.18 (1.08; 1.28)
**Abdominal obesity**^e^						
Prevalence (SE)	41.2 (2.2)	40.1 (2.1)	40.0 (2.3)	41.7 (2.4)		
Crude, *OR (95% CI)*	1	0.96 (0.75; 1.23)	0.95 (0.74; 1.23)	1.02 (0.79; 1.33)	0.903	1.01 (0.94; 1.07)
Model 1^b^, *OR (95% CI)*	1	0.96 (0.74; 1.24)	0.96 (0.75; 1.27)	1.27 (0.96; 1.69)	0.132	1.05 (0.98; 1.13)
Model 2^c^, *OR (95% CI)*	1	1.00 (0.77; 1.30)	0.99 (0.76; 1.29)	1.34 (1.00; 1.79)	0.076	1.06 (0.99; 1.14)

^a^ Quarters of proportion of ultra-processed foods in total energy intake. Sex specific cut-offs for quarters of ultra-processed food consumption were 36.3%, 51.0%, 61.1% and 76.2% in men and 35.2%, 50.4%, 60.1% and 73.1% in women.

^b^ Adjusted for sex, age, ethnicity, region, survey year, and social class occupation.

^c^ Adjusted for Model 1 + physical activity (time spent at moderate or vigorous physical activity) + smoking + sleep duration + following a special diet.

^d^ Defined as Body Mass Index ≥30 kg/m^2^ (World Health Organization, 2003).

^e^ Defined as waist circumference ≥102/88 cm for men and women, respectively (World Health Organization, 2008).

^α^ p value for linear trend across quartile of dietary contribution of ultra-processed foods.

The analyses stratified by sex are shown in Table 4. In women, the crude prevalence of obesity increased from 21.8% in the first to 33.3% in the fourth quartile of ultra-processed food consumption; while the crude prevalence of abdominal obesity increased from 45.7% to 47.3%, respectively. In multivariable analyses, the highest consumption of ultra-processed food was associated with 1.81 kg/m^2^ higher BMI (95%CI 0.81–2.81), 2.82 cm WC (95%CI 0.80–4.85) and double the odds for being obese (OR = 2.09, 95% CI 1.37–3.20), compared with the lowest consumption. In men, the crude prevalence of obesity increased from 24.2% in the first to 30.1% in the last quartile of ultra-processed food consumption; while the crude prevalence of abdominal obesity increased from 35.1% to 36.5%, respectively. In multivariable analyses, the highest consumption of ultra-processed food was associated with 1.35 kg/m^2^ higher BMI (95%CI 0.42–2.26), 3.35 cm WC (95%CI 1.71–6.99), and 1.62 odds for being obese (95%CI 1.02–2.57). A significant linear trend was observed for the association between quartile of ultra-processed food consumption and the three outcomes (P<0.05) in women and men. In the full adjusted model, a 10% increase in the consumption of ultra-processed foods was associated with an increase in BMI, WC and prevalence of obesity in both sexes. No association was observed for odds of abdominal obesity in both sexes.

**Table 4:** Crude and adjusted analyses of the association between the dietary contribution of ultra-processed food and indicators of obesity among the UK population aged 19 years or over, stratified by sex (NDNS, 2008–16).

	Consumption of ultra-processed foods (% of total energy)					
	Quartile ^a^				p for trend ^α^	Continuous (10% increase in the consumption)
	1	2	3	4		
**BMI (kg/m**^**2**^**)**						
Men						
Mean (SE)	27.4 (0.3)	27.4 (0.3)	26.8 (0.4)	29.9 (0.5)		
Crude, *coef (95% CI)*	0	0.03 (-0.8; 0.85)	-0.53 (-1.57; 0.51)	0.56 (-0.53; 1.64)	0.554	0.14 (-0.12; 0.41)
Model 1^b^, *coef (95% CI)*	0	-0.02 (-0.77; 0.74)	-0.39 (-1.29; 0.51)	1.09 (0.09; 2.109)	0.073	0.25 (0.00; 0.50)
Model 2^c^, *coef (95% CI)*	0	0.08 (-0.63; 0.80)	-0.25 (-1.08; 0.59)	1.35 (0.42; 2.26)	0.013	0.30 (0.05; 0.54)
Women						
Mean (SE)	26.5 (0.3)	26.9 (0.3)	27.6 (0.4)	28.1 (0.5)		
Crude, *coef (95% CI)*	0	0.38 (-0.5; 1.27)	1.07 (0.11; 2.03)	1.62 (0.49; 2.76)	0.002	0.43 (0.16; 0.71)
Model 1^b^, *coef (95% CI)*	0	0.30 (-0.56; 1.16)	0.97 (-0.01; 1.95)	1.72 (0.65; 2.78)	0.001	0.44 (0.17; 0.72)
Model 2^c^, *coef (95% CI)*	0	0.50 (-0.31; 1.31)	0.87 (-0.05; 1.80)	1.81 (0.81; 2.81)	<0.001	0.44 (0.19; 0.68)
**Waist circumference (cm)**						
Men						
Mean (SE)	97.4 (1.0)	97.8 (0.9)	96.0 (1.2)	98.6 (1.4)		
Crude, *coef (95% CI)*	0	0.52 (-2.12; 3.15)	-1.37 (-4.45; 1.71)	1.28 (-2.04; 4.6)	0.746	0.39 (-0.47; 1.26)
Model 1^b^, *coef (95% CI)*	0	0.18 (-2.04; 2.39)	-0.51 (-2.99; 1.96)	3.80 (1.01; 6.60)	0.016	0.95 (0.19; 1.71)
Model 2^c^, *coef (95% CI)*	0	0.50 (-1.65; 2.66)	-0.11 (-2.47; 2.25)	3.35 (1.71; 6.99)	0.003	1.04 (0.31; 1.77)
Women						
Mean (SE)	87.3 (0.7)	87.3 (0.8)	89.0 (0.9)	89.1 (1.0)		
Crude, *coef (95% CI)*	0	0.00 (-2.12; 2.12)	1.74 (-0.54; 4.02)	1.75 (-0.54; 4.05)	0.056	0.56 (0.02; 1.1)
Model 1^b^, *coef (95% CI)*	0	-0.13 (-2.09; 1.82)	1.61 (-0.59; 3.81)	2.58 (0.41; 4.74)	0.008	0.68 (0.15; 1.20)
Model 2^c^, *coef (95% CI)*	0	0.26 (-1.63; 1.15)	1.56 (-0.54; 3.69)	2.82 (0.80; 4.85)	0.004	0.71 (0.23; 1.20)
**Obesity** ^d^						
Men						
Prevalence (SE)	24.2 (2.8)	21.9 (2.6)	26.9 (3.2)	30.1 (3.7)		
Crude, *OR (95% CI)*	1	0.88 (0.57; 1.36)	1.15 (0.74; 1.79)	1.35 (0.85; 2.13)	0.106	1.12 (1.01; 1.26)
Model 1^b^, *OR (95% CI)*	1	0.81 (0.52; 1.25)	1.09 (0.70; 1.71)	1.42 (0.89; 2.25)	0.067	1.14 (1.02; 1.28)
Model 2^c^, *OR (95% CI)*	1	0.84 (0.54; 1.33)	1.20 (0.77; 1.87)	1.62 (1.02; 2.57)	0.015	1.18 (1.04; 1.33)
Women						
Prevalence (SE)	21.8 (2.4)	27.6 (2.7)	27.8 (2.8)	33.3 (3.0)		
Crude, *OR (95% CI)*	1	1.36 (0.93; 2.01)	1.38 (0.94; 2.04)	1.79 (1.22; 2.61)	0.004	1.14 (1.03; 1.26)
Model 1^b^, *OR (95% CI)*	1	1.36 (0.91; 2.03)	1.38 (0.91; 2.09)	1.93 (1.27; 2.93)	0.003	1.16 (1.04; 1.30)
Model 2^c^, *OR (95% CI)*	1	1.51 (1.00; 2.28)	1.37 (0.89; 2.10)	2.09 (1.37; 3.20)	0.002	1.17 (1.05; 1.30)
**Abdominal obesity** ^e^						
Men						
Prevalence (SE)	35.1 (3.1)	36.8 (3.0)	31.3 (3.2)	36.5 (3.6)		
Crude, *OR (95% CI)*	1	1.08 (0.74; 1.57)	0.84 (0.57; 1.25)	1.06 (0.71; 1.59)	0.894	1.01 (0.91; 1.11)
Model 1^b^, *OR (95% CI)*	1	1.00 (0.68; 1.46)	0.87 (0.59; 1.30)	1.37 (0.90; 2.08)	0.244	1.06 (0.95; 1.18)
Model 2^c^, *OR (95% CI)*	1	1.03 (0.70; 1.52)	0.91 (0.61; 1.35)	1.44 (0.95; 2.20)	0.162	1.07 (0.96; 1.19)
Women						
Prevalence (SE)	45.7 (2.9)	43.4 (2.9)	48.7 (3.0)	47.3 (3.1)		
Crude, *OR (95% CI)*	1	0.91 (0.66; 1.27)	1.13 (0.81; 1.57)	1.07 (0.76; 1.49)	0.457	1.04 (0.96; 1.13)
Model 1^b^, *OR (95% CI)*	1	0.89 (0.63; 1.26)	1.12 (0.79; 1.60)	1.21 (0.84; 1.74)	0.208	1.06 (0.97; 1.16)
Model 2^c^, *OR (95% CI)*	1	0.94 (0.66; 1.33)	1.12 (0.78; 1.61)	1.25 (0.86; 1.81)	0.185	1.06 (0.97; 1.17)

^a^ Quarters of proportion of ultra-processed foods in total energy intake. Cut-offs for quarters of ultra-processed food consumption were 36.3%, 51.0%, 61.1% and 76.2% in men and 35.2%, 50.4%, 60.1% and 73.1% in women.

^b^ Adjusted for sex, age, ethnicity, region, survey year, and social class occupation.

^c^ Adjusted for Model 1 + physical activity (time spent at moderate or vigorous physical activity) + smoking + sleep duration + following a special diet.

^d^ Defined as Body Mass Index ≥30 kg/m^2^ (World Health Organization, 2003).

^e^ Defined as waist circumference ≥102/88 cm for men and women, respectively (World Health Organization, 2008).

^α^ p value for linear trend across quartile of dietary contribution of ultra-processed foods.

In the sensitivity analysis, additional adjustment for total energy intake and for consumption of fruits and vegetables had little effect on the magnitude of the associations (S1 Table).

## Discussion

Findings from this nationally representative cross-sectional sample of UK adults show that higher consumption of ultra-processed food is associated with greater BMI, WC and odds of being obese. We identified a dose response relationship with a 10% increase in the consumption of ultra-processed foods being associated with a 18% and 17% increase in the prevalence of obesity in men and women, respectively.

Our results corroborate the findings from large, population-based cross-sectional and cohort studies that the consumption of ultra-processed foods is associated with obesity [16,20–22, 34], regardless of the contribution of these foods to the total diet (ranging from 25% in Brazil to 56% in the United States). Conversely, one study using data from the 2008–2012 UK NDNS found no association between relative energy intake from ultra-processed and moderately processed foods combined and body weight [23]. We point out that the food classification used by the mentioned study displays important differences with the one applied in our own that should be considered. More importantly, the group of processed foods, which include cheeses, vegetables and legumes preserved in brine, cured/canned meat and fish (but not sausage), were grouped together with ultra-processed foods. One analysis in Brazil showed that household purchase of ultra-processed foods was associated with greater prevalence of obesity, whereas processed foods were not [24]. Using ultra-processed foods as a specific group, rather than grouped with processed foods, may justify the difference in the findings from our study compared to other UK study [23].

Several mechanisms may explain the relationship between higher consumption of ultra-processed foods and obesity. Studies have shown strong associations between consumption of ultra-processed foods and dietary nutrient profiles known to increase the risk of several diet-related chronic diseases [8,11–13]. Diets based on these foods are energy-dense, high in free sugars, saturated and trans fats, and depleted in protein, fibre and most micronutrients [8,11–13]. A recent randomized controlled study of 20 healthy adults showed that diets high in ultra-processed foods increased dietary content of carbohydrates and fats, energy intake, body weight and fat gain, compared with a control diet matched in calories, sugar, fibre, and macronutrients [18].

These findings suggest that ultra-processed foods appear to promote overconsumption although the mechanism is not clear. Other characteristics related to the foods and/or the industrial processes, such as the greater deconstruction of the original food matrix and the cosmetic additives added to these products, have been associated with changes in the composition and metabolic behaviour of the gut microbiota that promote inflammatory diseases [35–38], with potential important implications for body weight and adiposity [39]. Moreover, ultra-processed foods are typically packaged in plastics, and several plasticizers such as bisphenol A have been shown to be associated with obesity [40,41].

The potential differences between sexes in the association between ultra-processed food consumption and obesity are supported by previous studies conducted in Brazil and the United States [20,21]. The reasons for the observed sex differences are unclear but may be related to the fact that women are more predisposed to adverse metabolic effects of rapidly digested, carbohydrate-rich foods than men [42]. Moreover, there are known gender difference in the type of ultra-processed foods consumed, with sweetened products more likely to be consumed among women (see Supporting Information–S2 Table). Therefore, this higher consumption of ultra-processed foods rich in sugars, in combination with the higher sensitivity to the hyper-glycaemic effects of these foods [43,44], might explain larger effects of ultra-processed foods on adiposity in women. Finally, the absence of a positive association between ultra-processed food consumption and abdominal obesity may be due to overall reduced statistical power consequence of dichotomizing a continuous outcome variable [45].

Our study has several strengths. We used the NOVA food classification system, which has been recognised as a valid tool for nutrition and public health research and policy [4, 46]. We also used the most updated version of NOVA that classifies processed foods and ultra-processed foods separately. In addition, we used data from the NDNS, a large and nationally representative sample of the UK population, increasing external validity of results. Notably, the disaggregation of dishes into underlying ingredients provided a more precise estimate of the dietary contributions of ultra-processed foods. Moreover, NDNS uses a high-quality dietary assessment method which provides detailed analysis of different foods consumed, which considered variations in consumption between different days of the week, as well as the seasonal variation.

Some limitations should be noted. Although food diaries are recognised to be one of the most comprehensive methods for assessing dietary intake, underreporting of some foods (particularly unhealthy foods) is a potential issue. Previous studies suggest that individuals with obesity may underreport consumption of foods with caloric sweeteners [47] such as desserts and sweet baked goods [48,49]. This social desirability bias may lead to underestimation of the dietary contribution of ultra-processed foods or dilution of the association between ultra-processed food consumption and adiposity. Notwithstanding, NDNS data were obtained through optimal methods for collecting dietary intake [50], which helped to reduce missing information. Though NDNS collects limited information indicating food processing (i.e., product brands and place of meals), for a small number of specific food items such as pizza there was insufficient information for classification purposes. In those cases, we choose the most commonly consumed alternative (culinary preparation or manufactured product) [8,30]. Finally, due to the cross-sectional nature of our data, reverse causality cannot be excluded. However, these findings are consistent with limited prospective data that reported consumption of ultra-processed foods and subsequent excess weight in two cohort studies [16, 35]. Due to the observational nature of our study, residual confounding cannot be ruled out.

The analyses presented here suggest that actions to reduce the consumption of ultra-processed foods could produce important public health benefits. Brazil [51], Uruguay [52] and Canada [53] have included the concept of ultra-processed foods in their food-based dietary guidelines. Recently, France set a goal of 20% reduction in consumption of ultra-processed foods by 2022 [54]. Actions to reduce consumption of some categories of ultra-processed foods has been also adopted in some countries. For instance, Mexico introduced fiscal measures, such as a tax on sugar-sweetened beverages and junk food, and studies already demonstrated a significant decline in the purchases of those products [55,56]. Voluntary agreements between industry and government have been shown repeatedly to be ineffective in improving public health [6]. This is confirmed by recent UK experience where the early stages of the government’s sugar reduction programme, which challenged the food industry to voluntarily cut sugar in some products, has produced only slow progress toward proposed targets [57]. Therefore, more radical measures that change the availability, price and marketing of products know to affect health is needed.

In conclusion, our findings suggest that ultra-processed foods may be contributing to the high rates of obesity in the UK and supports previous findings from Brazil, United States, Canada, and Spain. Several strategies should be employed to achieve necessary reductions in ultra-processed food consumption, including adequate food labelling, advertising regulation, and pricing policies. In addition, future dietary guidelines for the UK population should take food processing into account.

## Supporting information

S1 Table Crude and adjusted analyses of the association between the dietary contribution of ultra-processed food and indicators of obesity among the UK population aged 19 years or over, additional adjustment (NDNS, 2008–16). (DOC)

S2 Table Distribution of type of ultra-processed foods according to sex. UK population aged 19 years or over (2008–16).
(DOC)
