# Supplementary material for: Ultra-processed food consumption and indicators of obesity in the United Kingdom population (2008-2016)
Source: PLoS One. 2020 May 1;15(5):e0232676. doi: 10.1371/journal.pone.0232676 (PMC7194406; doi:10.1371/journal.pone.0232676)
Supplement: S1 Table — (DOC) [file pone.0232676.s001.doc]

| **S1 Table. Crude and adjusted analyses of the association between the dietary contribution of ultra-processed food and indicators of obesity among the UK population aged 19 years or over, additional adjustment (NDNS, 2008─16).** | | | | | | |
| --- | --- | --- | --- | --- | --- | --- |
|  | **Consumption of ultra-processed foods (% of total energy)** | | | | | |
|  | **Quartile^a^** | | | | **p for trend ^α^** | **Continuous (10% increase in the consumption)** |
|  | **1** | **2** | **3** | **4** |
|  | *Coefficient (95% CI)* | | | | | |
| **BMI (kg/m^2^)** |  |  |  |  |  |  |
| Total |  |  |  |  |  |  |
| Model 1 ^b^ | 0 | 0.41 (-0.14; 0.95) | 0.36 (-0.26; 0.99) | 1.67 (0.97; 2.37) | <0.001 | 0.38 (0.20; 0.55) |
| Model 2 ^c^ | 0 | 0.36 (-0.19; 0.91) | 0.31 (-0.33; 0.94) | 1.59 (0.87; 2.30) | <0.001 | 0.36 (0.18; 0.55) |
| Men |  |  |  |  |  |  |
| Model 1 ^b^ | 0 | 0.11 (-0.61; 0.84) | -0.23 (-1.07; 0.62) | 1.36 (0.43; 2.29) | 0.012 | 0.31 (0.06; 0.55) |
| Model 2 ^c^ | 0 | 0.04 (-0.68; 0.76) | -0.31 (-1.15; 0.52) | 1.24 (0.28; 2.20) | 0.031 | 0.27 (0.01; 0.53) |
| Women |  |  |  |  |  |  |
| Model 1 ^b^ | 0 | 0.50 (-0.31; 1.31) | 0.88 (-0.05; 1.81) | 1.81 (0.81; 2.81) | <0.001 | 0.44 (0.20; 0.68) |
| Model 2 ^c^ | 0 | 0.51 (-0.30; 1.33) | 0.89 (-0.06; 1.85) | 1.84 (0.81; 2.87) | <0.001 | 0.42 (0.19; 0.72) |
| **Waist circumference (cm)** |  |  |  |  |  |  |
| Total ^b^ |  |  |  |  |  |  |
| Model 1 ^b^ | 0 | 0.61 (-0.82; 2.04) | 0.66 (-0.92; 2.25) | 3.67 (1.92; 5.42) | <0.001 | 0.87 (0.41; 1.34) |
| Model 2 ^c^ | 0 | 0.39 (-1.04; 1.83) | 0.37 (-1.24; 1.97) | 3.21 (1.40; 5.00) | 0.001 | 0.76 (0.26; 1.26) |
| Men |  |  |  |  |  |  |
| Model 1 ^b^ | 0 | 0.49 (-1.66; 2.65) | -0.12 (-2.48; 2.25) | 3.35 (1.72; 6.97) | 0.003 | 1.04 (0.32; 1.77) |
| Model 2 ^c^ | 0 | 0.36 (-1.79; 2.51) | -0.33 (-2.70; 2.04) | 3.99 (1.27; 6.70) | 0.009 | 0.96 (0.19; 1.74) |
| Women |  |  |  |  |  |  |
| Model 1 ^b^ | 0 | 0.27 (-2.62; 2.15) | 1.58 (-0.54; 3.70) | 2.83 (0.80; 4.86) | 0.004 | 0.72 (0.23; 1.20) |
| Model 2 ^c^ | 0 | 0.09 (-1.81; 2.00) | 1.35 (-0.80; 3.50) | 2.48 (0.37; 4.59) | 0.013 | 0.63 (0.12; 1.15) |
|  |  |  |  |  |  |  |
|  | *Odds ratios (95% CI)* | | | | | |
| **Obesity** ^c^ |  |  |  |  |  |  |
| Total |  |  |  |  |  |  |
| Model 1 ^b^ | 1 | 1.20 (0.88; 1.22) | 1.31 (0.97; 1.78) | 1.91 (1.39; 2.62) | <0.001 | 1.18 (1.08; 1.28) |
| Model 2 ^c^ | 1 | 1.18 (0.87; 1.60) | 1.29 (0.95; 1.76) | 1.85 (1.34; 2.57) | <0.001 | 1.17 (1.07; 1.28) |
| Men |  |  |  |  |  |  |
| Model 1 ^b^ | 1 | 0.86 (0.54; 1.35) | 1.21 (0.78; 1.89) | 1.63 (1.02; 2.60) | 0.015 | 1.18 (1.04; 1.33) |
| Model 2 ^c^ | 1 | 0.82 (0.52; 1.30) | 1.15 (0.73; 1.83) | 1.51 (0.94; 2.45) | 0.038 | 1.16 (1.02; 1.32) |
| Women |  |  |  |  |  |  |
| Model 1 ^b^ | 1 | 1.50 (0.99; 2.28) | 1.37 (0.89; 2.10) | 2.09 (1.36; 3.19) | 0.002 | 1.17 (1.05; 1.30) |
| Model 2 ^c^ | 1 | 1.54 (1.02; 2.32) | 1.40 (0.91; 2.16) | 2.18 (1.41; 3.35) | 0.002 | 1.18 (1.05; 1.33) |
| **Abdominal obesity** ^d^ |  |  |  |  |  |  |
| Total |  |  |  |  |  |  |
| Model 1 ^b^ | 1 | 1.00 (0.77; 1.30) | 0.99 (0.76; 1.30) | 1.34 (1.00; 1.79) | 0.076 | 1.07 (0.99; 1.14) |
| Model 2 ^c^ | 1 | 0.98 (0.75; 1.28) | 0.97 (0.74; 1.26) | 1.28 (0.96; 1.73) | 0.155 | 1.05 (0.98; 1.14) |
| Men |  |  |  |  |  |  |
| Model 1 ^b^ | 1 | 1.03 (0.70; 1.53) | 0.91 (0.61; 1.35) | 1.44 (0.95; 2.20) | 0.164 | 1.07 (0.96; 1.19) |
| Model 2 ^c^ | 1 | 1.01 (0.68; 1.51) | 0.89 (0.60; 1.32) | 1.39 (0.90; 2.15) | 0.236 | 1.06 (0.95; 1.18) |
| Women |  |  |  |  |  |  |
| Model 1 ^b^ | 1 | 0.94 (0.66; 1.34) | 1.12 (0.78; 1.62) | 1.25 (0.86; 1.82) | 0.180 | 1.07 (0.98; 1.18) |
| Model 2 ^c^ | 1 | 0.93 (0.65; 1.32) | 1.10 (0.76; 1.60) | 1.22 (0.83; 1.80) | 0.238 | 1.07 (0.97; 1.18) |
| ^a^ Quarters of proportion of ultra-processed foods in total energy intake. Cut-offs for quarters of ultra-processed food consumption were 36.3%, 51.0%, 61.1% and 76.2% in men and 35.2%, 50.4%, 60.1% and 73.1% in women. | | | | | | |
| ^b^ Models adjusted for sex, age, ethnicity, region, survey year, social class occupation, physical activity (time spent at moderate or vigorous physical activity) + smoking + sleep duration + following a special diet + total energy intake. | | | | | | |
| ^c^ Models adjusted for sex, age, ethnicity, region, survey year, social class occupation, physical activity (time spent at moderate or vigorous physical activity) + smoking + sleep duration + following a special diet + fruit and vegetable consumption. | | | | | | |
| ^c^ Defined as Body Mass Index ≥30 kg/m^2^ (World Health Organization, 2003). | | | | | |  |
| ^d^ Defined as waist circumference ≥102/88 cm for men and women, respectively (World Health Organization, 2008). | | | | | | |
| ^α^ p value for linear trend across quartile of dietary contribution of ultra-processed foods. | | | | | |  |
