# Supplementary material for: Ultra-processed food consumption and indicators of obesity in the United Kingdom population (2008-2016)
Source: PLoS One. 2020 May 1;15(5):e0232676. doi: 10.1371/journal.pone.0232676 (PMC7194406; doi:10.1371/journal.pone.0232676)
Supplement: S2 Table — UK population aged 19 years or over (2008–16). (DOC) [file pone.0232676.s002.doc]

| **S2 Table. Distribution of type of ultra-processed foods according to sex. UK population aged 19 years or over (2008-16).** | | | |
| --- | --- | --- | --- |
| **Ultra-processed foods** | **% of energy intake from ultra-processed food** (*mean)* | | **P value** |
|  | **Men** | **Women** |  |
| Ultra-processed breads | 21.6 | 20.4 | 0.040 |
| Packaged pre-prepared meals ^a^ | 14.1 | 13.5 | 0.351 |
| Breakfast cereals | 7.9 | 8.5 | 0.142 |
| Sausage and other reconstituted meat products | 7.2 | 6.1 | 0.002 |
| Confectionary | 5.5 | 5.8 | 0.399 |
| Biscuits | 5.4 | 6.8 | <0.001 |
| Pastries, buns, and cakes | 5.8 | 6.1 | 0.419 |
| Industrial chips (French fries) | 4.6 | 4.1 | 0.089 |
| Soft drinks, fruit drinks and fruit juices | 3.9 | 3.4 | 0.136 |
| Milk-based drinks | 2.8 | 4.6 | <0.001 |
| Packaged salty snacks | 3.0 | 2.8 | 0.248 |
| Industrial pizza | 2.8 | 2.2 | 0.061 |
| Margarine and other spreads | 4.2 | 3.6 | 0.006 |
| Sauces, dressing and gravies | 4.0 | 5.0 | <0.001 |
| Industrial desserts | 1.4 | 1.8 | 0.036 |
| Other ultra-processed foods ^b^ | 5.9 | 5.3 | 0.155 |
| **Total** | **100** | **100** |  |
| ^a^ Including frozen and shelf-stable dishes and canned soups. | |  |  |
| ^b^ Including baked beans, meat alternatives, soy and others drinks as milk substitutes, and distilled alcoholic drink.  In blue: sweetened products | | | |
